# Editorial: The Bidirectional Communication Between Neurons and Immune Cells in the Development of Psychiatric, Neurological and Immune-Mediated Disorders

**DOI:** 10.3389/fimmu.2021.781151

**Published:** 2021-10-07

**Authors:** Antonietta Gentile, Fulvio D’Acquisto, Gordana Leposavić

**Affiliations:** ^1^ Synaptic Immunopathology Lab, IRCCS San Raffaele Pisana, Rome, Italy; ^2^ School of Life Science, University of Roehampton, London, United Kingdom; ^3^ Faculty of Pharmacy, Department of Pathobiology, University of Belgrade, Belgrade, Serbia

**Keywords:** neuroimmune axis, autoimmunity, T cells, microglia and macrophages, animal models

The capillary distribution of neuronal terminals and immune cells throughout the body allows the nervous and immune systems to interact and influence each other in response to different external stimuli. Such interaction is supported by the expression of receptors for cytokines and other immune mediators on neuronal surface, and by the expression of neurotransmitter receptors on adaptive and innate immune cells. The constitutive release of cytokines by glial cells and T cells residing in the meningeal space, regulate central nervous system (CNS) neuronal activity (1, 2). Likewise, peripheral sensory neurons respond to inflammatory mediators locally released by tissue-residing cells of the innate immune system (3). Dysfunctional communication between neurons and immune cells is increasingly acknowledged to play a role in neurological as well as non-neurological inflammatory and autoimmune disorders. This Research Topic includes three reviews and five original research articles, encompassing both inflammatory and autoimmune disorders and providing novel data and hints for future research in neuroimmunology.

The differential expression of α- and β2-adrenergic receptors on innate (macrophages) and adaptive immune cells (T cells) has been proposed to shape the development of Rheumatoid Arthritis (RA), an autoimmune pathology of the joints characterized by overactive sympathetic activity (4). Bellinger et al. have shown in a rat model of RA (the adjuvant-induced arthritis) that the combined administration of the α-adrenergic inhibitor phentolamine and the β2-adrenergic agonist terbutaline at the onset of the disease provided a beneficial effect on joint pathology by reducing the infiltration of inflammatory lymphocytes. Zhang and He provide an overview of the contribution of nociceptive neurons to the pathogenesis of psoriasis, a chronic inflammatory disease of the skin. The authors highlighted the contribution of Transient Receptor Potential (TRP) channels expressed on nociceptive neuronal terminals and T-helper 17 (Th17) cells in establishing a vicious circle that supports two classical symptoms of psoriasis: pruritus and hyperalgesia. The article also provides evidence for the need of further investigations on the use of anti-IL-17 antibodies and Botox therapies in the treatment of psoriasis.

Brain antigen-specific autoimmune disorders include Multiple Sclerosis (MS) as well as anti-AMPA and anti-NMDA receptors encephalitis. Noteworthy, glutamate receptor antibodies have been detected in the cerebrospinal fluid of patients affected by neuropsychiatric disorders and pathological disorders classically regarded as proteinopathies. These include frontotemporal dementia (5), making hard to decipher whether these autoantibodies have a causal role or represent an epiphenomenon in the above-mentioned pathologies. In this regard, Cisani et al. showed that the presence GluA2 and GluA3 antibody complexes increase the release of glutamate from isolated cortical synaptosomes by favoring the membrane insertion of AMPA autoreceptors. In addition, the presence of GluA2 or GluA3 antigen-antibody complexes in neuronal endings facilitated the Cq1 complement-mediated glutamate release. These findings may provide an explanation for the symptoms related to excessive glutamatergic transmission in pathological conditions featured by the presence of anti-GluA2 and anti-GluA3 antibodies.

The study by Alrashdi et al. addressed the role of voltage gated sodium (Nav) channel isoform 1.6 (NaV1.6) in regulating the immune response. The function of these channels in axonal conductance is quite well established (6) while their role in immune cells has been only recently explored. Mice heterozygous for the gene encoding for NaV1.6 subjected to Experimental Autoimmune Encephalitis (EAE) showed a reduced inflammatory response and a decreased recruitment of myeloid cells in the optic nerve. Interestingly, the immunomodulatory effect of these channels was not limited to autoimmune inflammation. Indeed, administration of lipopolysaccharide (LPS) to the same mice showed a similar reduction in the amplitude of the inflammatory response – thus suggesting a wider role of these channels in the immune response of the host.

Two studies in this collection have focused on ischemic stroke. To address the contribution of different types of immune cells to the neurological outcome of this pathology, Liu and Sorooshyari sought to correlate neurological deficits with the number of splenic immune cells recruited into the brain following the Middle Cerebral Artery Occlusion (MCAO; a murine model of stroke). The results of these analyses suggested that microglia and splenic T and B cells play a protective role while macrophages and neutrophils exacerbate the motor complications observed in this model of disease. Using the same experimental model, Zhu et al. proposed that administration of a selective JAK 1/2 inhibitor Ruxolitinib - a potent tumor and immune suppressor - might be beneficial for recovery from stroke. The study shows that targeting the JAK2/STAT3 pathway improved neurological deficits, neuropathological damage as infarct area, and reduced the number of infiltrating macrophages, B and T cells, by inhibiting the expression of pro-inflammatory cytokines and NLRP3 inflammasome.

Finally, two reviews addressed the role of inflammation in neurodegenerative diseases. Mancini et al. analyzed the contribution of astrocytes and microglia to synaptic plasticity in the striatum. This is a core area of the basal ganglia that plays a key role in a wide range of motor, cognitive and affective behaviors. The authors discuss how an aberrant activation of the immune system paired with high levels of pro-inflammatory cytokines in the basal ganglia contributes to alterations in synaptic plasticity. Consistent with this, patients suffering from Parkinson’s Disease (PD) have been reported to present a heightened inflammatory response in these areas of the brain.

The minireview by Stojić-Vukanić et al. identify CD8+ T cells as a common player in the neurocognitive impairment that features Alzheimer’s Disease (AD) and MS. The article provides neuropathological evidence supporting the presence of these cells in the brain of AD and MS patients and discuss the putative mechanisms by which CD8+ T cells cause neuronal damage and cognitive deficits. As the authors suggested, further investigations in this area of research might lead to the discovery of novel dual therapies that can provide beneficial effects for both AD and MS.

To conclude, we are aware that the articles included in this Research Topic raise many intriguing questions more than definite answers. We are nonetheless hopeful that our readers will be inspired by the research presented and consider the crosstalk between brain and immune system as a novel exciting area of investigation.

## Author Contributions

All authors contributed to writing the editorial and approved it for publication.

## Funding

AG acknowledges support from the Italian Ministry of Health (GR-2018-12366154).

## Conflict of Interest

The authors declare that the research was conducted in the absence of any commercial or financial relationships that could be construed as a potential conflict of interest.

## Publisher’s Note

All claims expressed in this article are solely those of the authors and do not necessarily represent those of their affiliated organizations, or those of the publisher, the editors and the reviewers. Any product that may be evaluated in this article, or claim that may be made by its manufacturer, is not guaranteed or endorsed by the publisher.
